# Malakoplakia of the appendix in a young healthy adult: a case report and literature review

**DOI:** 10.1093/jscr/rjac357

**Published:** 2022-08-04

**Authors:** Dario Pastena, Mauro Giambusso, Federica Castri, Angelo Eugenio Potenza, Paola Caprino, Franco Sacchetti, Luigi Sofo

**Affiliations:** Division of Abdominal Surgery, Department of Medical and Surgical Sciences, Fondazione Policlinico Universitario Agostino Gemelli IRCCS, Rome, Italy; Division of Abdominal Surgery, Department of Medical and Surgical Sciences, Fondazione Policlinico Universitario Agostino Gemelli IRCCS, Rome, Italy; Division of General Pathological Anatomy, Department of Women's and Child Health and Public Health Sciences, Fondazione Policlinico Universitario Agostino Gemelli IRCCS, Rome, Italy; Division of Abdominal Surgery, Department of Medical and Surgical Sciences, Fondazione Policlinico Universitario Agostino Gemelli IRCCS, Rome, Italy; Division of Abdominal Surgery, Department of Medical and Surgical Sciences, Fondazione Policlinico Universitario Agostino Gemelli IRCCS, Rome, Italy; Division of Abdominal Surgery, Department of Medical and Surgical Sciences, Fondazione Policlinico Universitario Agostino Gemelli IRCCS, Rome, Italy; Division of Abdominal Surgery, Department of Medical and Surgical Sciences, Fondazione Policlinico Universitario Agostino Gemelli IRCCS, Rome, Italy

**Keywords:** malakoplakia, appendix, case report

## Abstract

Malakoplakia is a rare entity on inflammatory base that mostly occurs in immunocompromised individuals which is thought to be secondary to a bactericidal defect in macrophages. The genitourinary tract is typically affected. The appendix is a very rare localization. We report a case of malakoplakia in the appendix of a young healthy patient with a recent history of abdominal pain associated with diarrhea and nausea. The colonscopy and CT scan showed an extramucosal bumping mass pressing on the cecum and covered by normal mucosa. The patient underwent to laparoscopic appendectomy. The histology showed a malakoplakia of the appendix. Gastrointestinal localization of malakoplakia is often associated with preexisting diseases, which are probably responsible for an immune disorder underlying the etiopathogenesis of the disease. However, in our case, the patient had no comorbidities. Probably, a clinically unknown immune predisposition plays an important role. Further studies are needed to clarify this nexus.

## INTRODUCTION

Malakoplakia is an inflammatory reaction to organisms, which include bacteria, mycobacteria, fungi and occasionally parasites. Usually makes its presence known as a papule, plaque or ulceration which generally occurs in the genitourinary tract; nevertheless, it has been described in almost all bodily organs.

Malakoplakia arises on average around 50 years of age; pediatric cases are rare [1]. Women are found to be affected more frequently than men.

Malakoplakia, from the Greek ‘malakos’, meaning soft, and ‘plakion’, meaning plaque, is the term that was coined by von Hanseman to describe the first human case in 1903, a postmortem bladder finding of a 66-year-old man who died of pulmonary tuberculosis, characterized by the microscopic relief of soft and elevated, yellow-to-brownish, lesions. Subsequent early cases of malakoplakia were all showed in the genitourinary tract [2].

Within the genitourinary tract, which remains the most affected site, the urinary bladder is the most frequent region of onset [3]. However, malakoplakia may involve other organs and structures, especially the gastrointestinal tract (GIT). Regarding the GIT, the tract most involved is the colon [4, 5]. Several studies in the literature describe the rectum as the first localization, followed by the sigma [6]. The appendix is a rare localization of the disease, with very few cases described in the literature. Cutaneous manifestations, which are less frequent, mainly affect the perineum [7].

We describe a case of malakoplakia of the appendix, probably the seventh reported in literature.

## CASE REPORT

A 33-year-old male presented himself to our clinic with a 1-year history of increasing abdominal pain, diarrhea and nausea.

The colonoscopy showed an extraluminal bumping mass pressing on the cecum of about 4 cm covered by regular mucosa compatible with gastrointestinal stromal tumor (GIST) or lipoma; it was not possible to identify the appendicular dimple.

The CT scan of the abdomen found a pseudonodular relatively inhomogeneous submucosal mass located by the median wall of the cecum, of about two centimeters in size with relatively thickened margins and fluid dense content. Apparent continuity with the appendicular lumen which appears relatively thinned and occupied by material of moderate density. The finding could refer to a locoregional inflammatory collection in continuity with the cecum and the appendicular lumen.

The patient was submitted to laparoscopic appendectomy, with and intraoperative finding of an intraluminal mass of the base of the appendix. A stapled section of the appendix with a partial inclusion of the wall of the cecum was performed. The patient was discharged 2 days after the surgery; no post-operative complications occurred.

The resected specimen consisted of 8-cm-long appendix with enlarged lumen, thickened walls for 1.5 cm. The cecum resection margin was free of disease.

**Figure 1:**
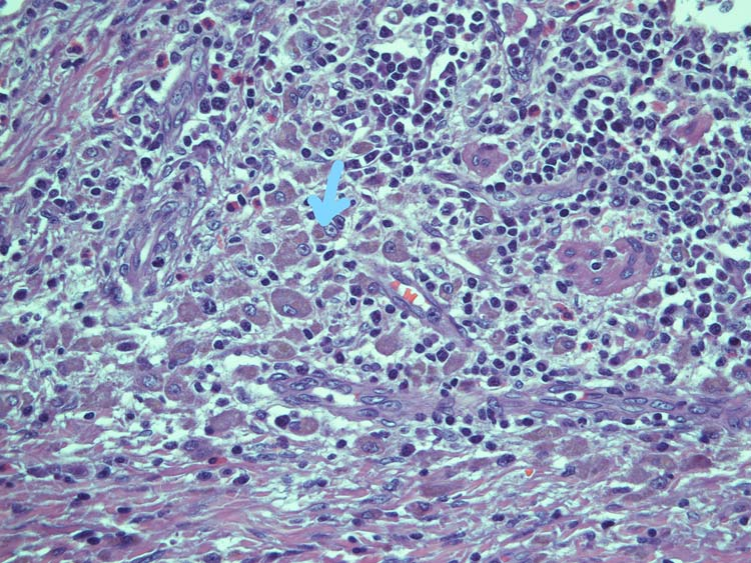
Submucosal cellular population is constituted by round histiocytic elements with a granular dense cytoplasm, containing typical eosinophilic bodies (arrow); haematoxylin and eosin, magnification ×20.

**Figure 2:**
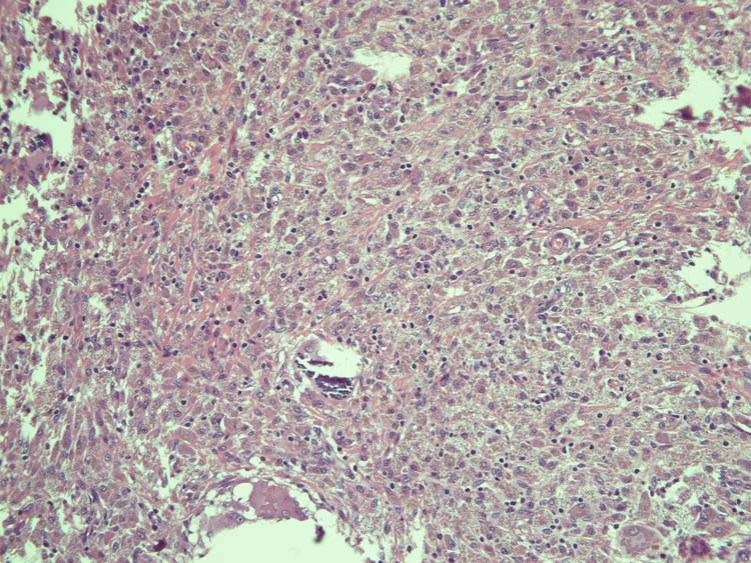
Submucosa is expanded and colonized by a dense histiocytic population (von Hansemann cells) and harboring microcalcifications; haematoxylin and eosin, magnification ×20.

**Figure 3:**
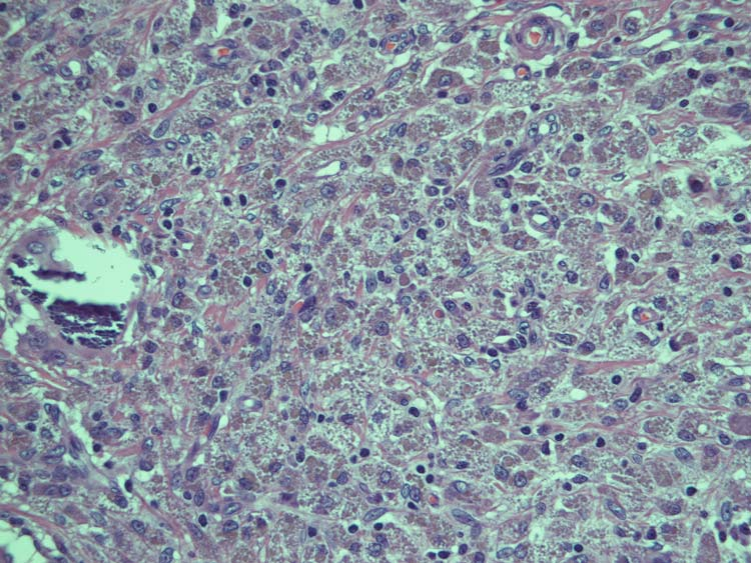
Intracellular and extracellular Michaelis–Gutmann bodies surrounded by inflammatory cell infiltrate of histiocytes; haematoxylin and eosin, magnification ×40.

**Figure 4:**
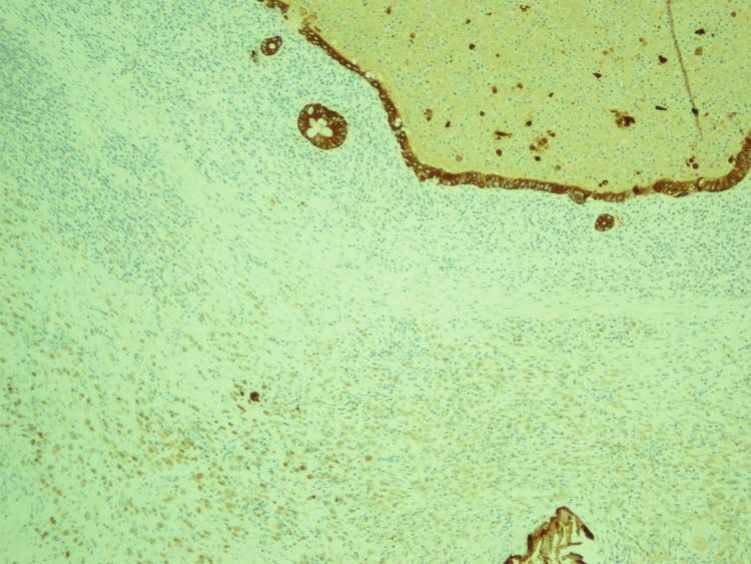
The most represented population is pancytokeratin negative, confirming its non-epitheliod and non-neoplastic nature; pancytokeratin, magnification ×10.

On microscopy, the appendix showed a thickening of his walls due to an accumulation of rounded elements with rich granular cytoplasm containing amorphous corpuscles and surrounded by microcalcifications, macrophage elements with adjacent de-epithelialized, ulcerated and eroded mucous tracts. No positive S100/pancytokeratin elements were found in the numerous sections examined. The morphological findings therefore indicated a form of malakoplakia (Figs 1–3), confirming its non-eptheliod and non-neoplastic nature (Fig. 4).

At 1, 3 and 6 months follow-up, the patient results in good condition.

## DISCUSSION

In 1970, W. M. Blackshear [8] described the first known case of malakoplakia of the appendix. Few other cases of malakoplakia of this site have been described subsequently [9, 10], among them singular is the association with Taenia eggs found by Jain *et al.* [11] in 2000. Patients with malakoplakia often have an immunodeficiency condition, such as concomitant neoplastic disease, autoimmune disease or organ transplant histories. Evident in the literature is the association with other granulomatous diseases, such as sarcoidosis [12] or tuberculosis [13], and with carcinomas, especially of the GIT [14]. Shaktawat *et al.* in 2008 [15] described a case of malakoplakia of the appendix associated with ulcerative colitis (UC), suggesting how an immune disorder related to UC itself may underlie this appendicular pathology.

Malakoplakia is thought to be due to a defect in the response to bacterial infection. Macrophages and monocytes show deficient phagolysosomal activity; they phagocytose bacteria but are unable to digest them completely. Partially digested bacteria accumulate in the cell cytoplasm and produce a granulomatous immune cell reaction. Diagnosis is not easy, as it can also run asymptomatically and be an entirely incidental finding. In cases with gastrointestinal involvement, the most common symptoms are abdominal pain, diarrhea, bleeding and dyspepsia. Recognizing malakoplakia is difficult because it is not associated with either specific clinical symptoms or characteristic imaging pictures. Diagnosis is based on endoscopy findings, which reveal the presence of yellowish vascularized plaques or white polypoid nodules. Confirmation is made by biopsy of the affected tissues. Histologic examination demonstrates the presence of von Hansemann cells, or rather histiocytes with small nuclei and acidophilic granular cytoplasm containing Michaelis–Gutmann bodies (calcium inclusions positive for Schiff’s periodic acid and von Kossa staining). This lesion is pathognomonic for malakoplakia.

Treatment is based on administration antibiotics capable of penetrating inside the cells, as fluoroquinolones and trimethoprim-sulfamethoxazole. The duration of therapy is not yet standardized. In pseudotumor forms, as in our case, surgical removal of lesions is indicated.

Gastrointestinal malakoplakia is often associated with diseases underlying immunosuppressive states. However, this association has only been hypothesized in the case of appendicular localization. Indeed, in our case, the patient, did not suffer from any known comorbidities, posing a not indifferent question mark on the etiopathogenesis of the condition. Given the patient’s young age, it cannot be ruled out that an unknown immune predisposition may have led to the development of appendicular malakoplakia. However, further studies are needed to better elucidate the predisposing factors for this condition.
